# Socioeconomic and labor characterization and prevalence of chronic disease in the Colombian population in the periods 2010, 2013, and 2016: A multiple correspondence analysis

**DOI:** 10.1590/1980-549720250036

**Published:** 2025-07-04

**Authors:** Francisco Palencia-Sánchez, Gustavo Antonio Bruges Morales, Martha Riaño-Casallas

**Affiliations:** I Pontificia Universidad Javeriana, School of Medicine – Bogotá, Colombia.; II Universidad Autónoma de Bucaramanga, School of Health Sciences – Floridablanca, Santander, Colombia.; III Universidad Nacional de Colombia, School of Economic Sciences – Bogotá, Colombia.

**Keywords:** Social determinants of health, Chronic disease, Working conditions, Public health, Multivariate analysis, Socioeconomic factors

## Abstract

**Objective:**

To analyze the relationship between the prevalence of chronic diseases and socioeconomic, demographic and occupational determinants in the Colombian population in 2010, 2013 and 2016. We sought to identify patterns of association between these variables and evaluate how they have evolved over time, with a particular emphasis on the implications for public health, especially in informal work contexts.

**Methods:**

We used longitudinal data from the Colombian Longitudinal Urban Survey, which covers socioeconomic strata 1 to 4. The sample includes both men and women and heads of households and spouses, aged 18 to 65 years. The variables of interest are classified into three categories: health, labor and sociodemographic factors. The main methodology used was Multiple Correspondence Analysis (MCA).

**Results:**

Three different labor groups were identified in the sample: formal, semi-formal and informal. The informal workers group showed a higher prevalence of chronic diseases compared to the other two groups. Labor category was the social determinant of greatest relevance in health variability.

**Conclusion:**

The study’s findings indicate that labor informality is associated with an elevated risk of developing chronic diseases in Colombia. These results highlight the necessity for policy interventions that prioritize enhancing working conditions as a strategy to achieve improved public health outcomes.

## INTRODUCTION

The interaction between workers’ health status and job performance significantly influences productivity and the functioning of health and pension systems worldwide. This relationship has led to numerous studies focusing on the economically active population (EAP), with particular emphasis on chronic diseases (CDs), which are defined by the World Health Organization^1^ as long-term conditions arising from a combination of genetic, physiological, environmental, and behavioral factors.

Studies indicate that a significant proportion of the working population is affected by one or more CDs, directly influencing decisions regarding disability pensions. These conditions have been shown to reduce work capacity by approximately 17.8 to 36.4%^2^. Mental, cardiovascular, and musculoskeletal disorders are frequently identified as leading causes of early withdrawal from the labor market. In Finland, 4,889 disability pension claims were recorded among workers aged 35 to 54 with mental health disorders^3^. This context, combined with the healthy worker effect, presents challenges in further investigating CDs within occupational settings. The healthy worker effect refers to the observed phenomenon in which workers tend to have lower mortality rates than the general population, as individuals with severe illnesses or disabilities are typically excluded from employment^4^. As a result, the seemingly lower prevalence of certain diseases among active workers is primarily attributed to selection bias. This effect partially obscures the actual relationship between employment and CDs, thereby limiting research in this area.

Traditionally, the impact of CDs has been analyzed from the perspective of the health system, involving stakeholders such as clinicians, insurers, and public policymakers. However, expanding this approach is essential to gain a more comprehensive understanding of the implications of CDs in the workplace, particularly through the examination of social determinants of health, with a specific focus on work^5^.

Global labor market trends indicate an increasing proportion of workers aged 55 to 64, with variations across economic sectors, carrying significant implications for occupational health^6^. In response, public policy initiatives have been proposed to promote the inclusion of older workers, who are at heightened risk of developing CDs^7,8^.

In Colombia, data indicate that the primary impact on workers’ health results from general illness rather than occupational illness. General illness is defined as a condition arising from a common disease or accident not related to occupational exposure^9^. According to the National Association of Industrialists, 65.3% of absenteeism cases in companies were attributed to general illness^10^. These figures, which pertain exclusively to formal workers, highlight the importance of considering various employment categories (salaried, self-employed, informal) and their respective associations with CDs.

In recent years, a global increase in the EAP has been observed. In the United States, the proportion of workers aged 55 to 64 is expanding at a faster rate than other age groups within the labor force, with this trend varying across economic sectors^11^.

In Colombia, the exhaustion of the “demographic dividend” and the growing number of individuals over the age of 40 within the EAP present challenges related to demographic and epidemiological transitions. These shifts may influence the incidence and prevalence of CDs, thereby impacting healthcare system costs^12,13,14^. Regarding causes of death in Colombia, CDs such as cardiovascular disease, chronic obstructive pulmonary disease, and stroke were identified in 2021 as major contributors, according to a global study on leading causes of mortality^15^. Furthermore, a report by the Bank of the Republic of Colombia indicates that workers’ health is primarily affected by general illnesses rather than occupational conditions^16^.

CDs are multifactorial conditions; therefore, multiple correspondence analysis (MCA) serves as a valuable tool for capturing this complexity by identifying homogeneous patterns^17^. The objective of this study was to analyze the prevalence of CDs and their association with socioeconomic and occupational variables in a sample of individuals monitored in Colombia during the years 2010, 2013, and 2016, using MCA

## METHODS

### Data source

The Colombian Longitudinal Survey (*Encuesta Longitudinal Colombiana* – ELCA) was used in its urban component, which has tracked 6,000 households longitudinally since 2010, with additional assessments conducted in 2013 and 2016. Designed by the Center for Economic Development Studies (*Centro de Estudios sobre Desarrollo Económico* – CEDE)^18^ of the School of Economics at Universidad de los Andes in Colombia, this survey enabled the collection of data on temporal changes in dynamics affecting population well-being. The sample is nationally representative of socioeconomic strata 1 to 4 and five geographic regions of Colombia, employing a probabilistic, stratified, multi-stage, and cluster-based sampling design^10^.

The significance of this study lies in its longitudinal design, which addresses the need in Colombia to strengthen prospective research on key populations for public policy decision-making. In this context, the strategy involved tracking the same group of individuals across the three waves of the survey.

### Methodology

The study focused on the economically active population, defined as individuals of working age who are either employed and/or actively seeking employment. For the analysis, household heads aged 18 to 65 were selected at baseline, with individuals under 18 excluded to avoid potential misclassification related to child labor^19,20,21^.

The variables analyzed include health, employment, and sociodemographic characteristics, as presented in Table 1. To ensure the reliability and validity of the study, the following were treated as confounding variables: *socioeconomic status*, given its influence on both employment opportunities and access to healthcare services; *age*, due to its impact on the selection of employment sectors and the prevalence of CDs; and *region*, as it may affect educational access and regional economic conditions.

**Table 1:** Descriptive summary (*) of the variables studied in the ELCA Survey for the periods 2010, 2013, and 2016.

Year	2010		2013		2016	
Characteristic	men	women	men	women	men	women
	*n=2,286*	*n=3,347*	*n=2,286*	*n=3,347*	*n=2,286*	*n=3,347*
**Self-reported chronic illness or pain**						
*No*	1,636 (71.6%)	2,150 (64.2%)	1,264 (55.3%)	1,560 (46.6%)	1,057 (46.2%)	1,229 (36.7%)
*Yes*	650 (28.4%)	1,197 (35.8%)	1,022 (44.7%)	1,787 (53.4%)	1,229 (53.8%)	2,118 (63.3%)
**Age (years)**						
*18-34*	560 (24.5%)	1,003 (30.0%)	393 (17.2%)	730 (21.8%)	240 (10.5%)	478 (14.3%)
*35-50*	1,033 (45.2%)	1,532 (45.8%)	996 (43.6%)	1,533 (45.8%)	958 (41.9%)	1,474 (44.0%)
*51-71*	693 (30.3%)	812 (24.3%)	897 (39.2%)	1,084 (32.4%)	1,088 (47.6%)	1,395 (41.7%)
**Marital status**						
*Married*	1,016 (44.4%)	1,134 (33.9%)	1,072 (46.9%)	1,167 (34.9%)	1,098 (48.0%)	1,176 (35.1%)
*Separated or divorced*	1,100 (48.1%)	1,277 (38.2%)	1,000 (43.7%)	1,137 (34.0%)	941 (41.2%)	1,054 (31.5%)
*Single*	84 (3.7%)	511 (15.3%)	125 (5.5%)	603 (18.0%)	153 (6.7%)	624 (18.6%)
*Widowed*	75 (3.3%)	269 (8.0%)	71 (3.1%)	266 (7.9%)	69 (3.0%)	286 (8.5%)
*In a partnership*	11 (0.5%)	152 (4.5%)	18 (0.8%)	174 (5.2%)	25 (1.1%)	207 (6.2%)
*Not informed*	0 (0.0%)	4 (0.1%)	0 (0.0%)	0 (0.0%)	0 (0.0%)	0 (0.0%)
**Position in the household**						
*Head of the household*	2,080 (91.0%)	1,259 (37.6%)	2,080 (91.0%)	1,259 (37.6%)	2,080 (91.0%)	1,259 (37.6%)
*Spouse*	206 (9.0%)	2,088 (62.4%)	206 (9.0%)	2,088 (62.4%)	206 (9.0%)	2,088 (62.4%)
**Region of the country**						
*Atlantic*	604 (26.4%)	816 (24.4%)	604 (26.4%)	816 (24.4%)	604 (26.4%)	816 (24.4%)
*Bogotá*	331 (14.5%)	509 (15.2%)	331 (14.5%)	509 (15.2%)	331 (14.5%)	509 (15.2%)
*Central*	405 (17.7%)	648 (19.4%)	405 (17.7%)	648 (19.4%)	405 (17.7%)	648 (19.4%)
*Eastern*	473 (20.7%)	691 (20.6%)	473 (20.7%)	691 (20.6%)	473 (20.7%)	691 (20.6%)
*Pacific*	473 (20.7%)	683 (20.4%)	473 (20.7%)	683 (20.4%)	473 (20.7%)	683 (20.4%)
**Ethnic group**						
*Afro-Colombian*	225 (9.8%)	275 (8.2%)	225 (9.8%)	275 (8.2%)	225 (9.8%)	275 (8.2%)
*White-Mixed race*	1,930 (84.4%)	2,879 (86.0%)	1,930 (84.4%)	2,879 (86.0%)	1,930 (84.4%)	2,879 (86.0%)
*Indigenous*	131 (5.7%)	193 (5.8%)	131 (5.7%)	193 (5.8%)	131 (5.7%)	193 (5.8%)
**Socioeconomic stratum of the household**						
*stratum-1*	745 (32.6%)	1,019 (30.4%)	748 (32.7%)	1,038 (31.0%)	723 (31.6%)	1,010 (30.2%)
*stratum-2*	912 (39.9%)	1,361 (40.7%)	907 (39.7%)	1,344 (40.2%)	931 (40.7%)	1,353 (40.4%)
*stratum-3*	534 (23.4%)	834 (24.9%)	547 (23.9%)	842 (25.2%)	526 (23.0%)	836 (25.0%)
*stratum-4*	95 (4.2%)	133 (4.0%)	84 (3.7%)	123 (3.7%)	106 (4.6%)	148 (4.4%)
**Educational level**						
*Primary*						
*No*	1,397 (61.1%)	2,095 (62.6%)	1,503 (65.7%)	2,271 (67.9%)	1,511 (66.1%)	2,293 (68.5%)
*Yes*	889 (38.9%)	1,252 (37.4%)	783 (34.3%)	1,076 (32.1%)	775 (33.9%)	1,054 (31.5%)
*Secondary*						
*No*	1,330 (58.2%)	1,918 (57.3%)	1,325 (58.0%)	1,933 (57.8%)	1,333 (58.3%)	1,976 (59.0%)
*Yes*	956 (41.8%)	1,429 (42.7%)	961 (42.0%)	1,414 (42.2%)	953 (41.7%)	1,371 (41.0%)
*Higher*						
*No*	1,892 (82.8%)	2,741 (81.9%)	1,817 (79.5%)	2,573 (76.9%)	1,813 (79.3%)	2,528 (75.5%)
*Yes*	394 (17.2%)	606 (18.1%)	469 (20.5%)	774 (23.1%)	473 (20.7%)	819 (24.5%)
**Type of employment**						
*Self-employed*	1,060 (46.4%)	1,086 (32.4%)	1,063 (46.5%)	1,235 (36.9%)	1,085 (47.5%)	1,249 (37.3%)
*Salaried*	986 (43.1%)	776 (23.2%)	979 (42.8%)	802 (24.0%)	898 (39.3%)	820 (24.5%)
*Other*	240 (10.5%)	1,485 (44.4%)	244 (10.7%)	1,310 (39.1%)	303 (13.3%)	1,278 (38.2%)
**Salary level**						
*Not informed*	1,215 (53.1%)	2,412 (72.1%)	1,534 (67.1%)	2,543 (76.0%)	1,587 (69.4%)	2,511 (75.0%)
*Low* LMMW	227 (9.9%)	395 (11.8%)	282 (12.3%)	483 (14.4%)	224 (9.8%)	452 (13.5%)
*Up to 2* LMMW	603 (26.4%)	394 (11.8%)	470 (20.6%)	321 (9.6%)	475 (20.8%)	384 (11.5%)
*More than 2* LMMW	241 (10.5%)	146 (4.4%)	0 (0.0%)	0 (0.0%)	0 (0.0%)	0 (0.0%)
**Type of contract**						
*Written fixed-term contract*	106 (4.6%)	249 (7.4%)	254 (11.1%)	193 (5.8%)	236 (10.3%)	256 (7.6%)
*Written indefinite-term contract*	965 (42.2%)	686 (20.5%)	531 (23.2%)	388 (11.6%)	537 (23.5%)	430 (12.8%)
*Verbal contract*	91 (4.0%)	24 (0.7%)	129 (5.6%)	142 (4.2%)	150 (6.6%)	170 (5.1%)
*Does not apply*	61 (2.7%)	115 (3.4%)	497 (21.7%)	631 (18.9%)	37 (1.6%)	56 (1.7%)
*No contract*	823 (36.0%)	788 (23.5%)	606 (26.5%)	654 (19.5%)	1,023 (44.8%)	1,157 (34.6%)
*NR*	240 (10.5%)	1,485 (44.4%)	269 (11.8%)	1,339 (40.0%)	303 (13.3%)	1,278 (38.2%)
**Economic Sector in which the individual works**						
*None*	240 (10.5%)	1,485 (44.4%)	269 (11.8%)	1,339 (40.0%)	303 (13.3%)	1,278 (38.2%)
*Primary*	232 (10.1%)	58 (1.7%)	237 (10.4%)	54 (1.6%)	204 (8.9%)	46 (1.4%)
*Secondary*	540 (23.6%)	320 (9.6%)	510 (22.3%)	312 (9.3%)	459 (20.1%)	213 (6.4%)
*Tertiary*	1,274 (55.7%)	1,484 (44.3%)	1,270 (55.6%)	1,642 (49.1%)	1,320 (57.7%)	1,810 (54.1%)
**Natural Gas Public Service**						
*No*	699 (30.6%)	1,032 (30.8%)	509 (22.3%)	750 (22.4%)	385 (16.8%)	567 (16.9%)
*Yes*	1,587 (69.4%)	2,315 (69.2%)	1,777 (77.7%)	2,597 (77.6%)	1,901 (83.2%)	2,780 (83.1%)
**Telephone Public Service**						
*No*	1,134 (49.6%)	1,640 (49.0%)	1,184 (51.8%)	1,727 (51.6%)	1,251 (54.7%)	1,807 (54.0%)
*Yes*	1,152 (50.4%)	1,707 (51.0%)	1,102 (48.2%)	1,620 (48.4%)	1,035 (45.3%)	1,540 (46.0%)
**Paternal history of disease**						
No	1,409 (61.6%)	1,994 (59.6%)	1,409 (61.6%)	1,994 (59.6%)	1,409 (61.6%)	1,994 (59.6%)
Yes	877 (38.4%)	1,353 (40.4%)	877 (38.4%)	1,353 (40.4%)	877 (38.4%)	1,353 (40.4%)
**Maternal history of disease**						
*No*	1,166 (51.0%)	1,629 (48.7%)	1,166 (51.0%)	1,629 (48.7%)	1,166 (51.0%)	1,629 (48.7%)
*Yes*	1,120 (49.0%)	1,718 (51.3%)	1,120 (49.0%)	1,718 (51.3%)	1,120 (49.0%)	1,718 (51.3%)

*Weighted sample estimates.

LMMW:Legal Minimum Monthly Wage

Each survey observation includes an expansion factor to ensure statistical representativeness. These factors were applied directly as weights in the calculation of proportions and subsequent analyses, using the survey package in R. For additional methodological details, refer to the user guide (https://datoscede.uniandes.edu.co/wp-content/uploads/Guia-Usuario.pdf)^22^.

### Descriptive statistics

A table was constructed to present the absolute frequencies and percentages of the categorical variables. This includes an assessment of the prevalence of CDs, the distribution of contract types and economic sectors, as well as sociodemographic characteristics such as educational level, socioeconomic status, and ethnicity, disaggregated by each year of the study (2010, 2013, and 2016). The descriptive tables were generated using the *compareGroups* package in R^23^.

For the calculations, functions from the survey package in R were employed, which automatically apply expansion factors as statistical weights “survey: analysis of complex survey samples,” R package version 4.4)^24^.

### Multiple correspondence analysis

MCA is a technique used to visualize patterns in large, high-dimensional categorical datasets^17^. In MCA, interpretation is guided by assessing the contribution of variables to each axis, using measures such as inertia, eigenvalues, and squared cosine (cos^2^). This study emphasizes the use of cos^2^ to evaluate the quality of graphical representation, thereby facilitating the identification of variables that are optimally projected within the factorial space. In MCA, the distance between categories of different variables is interpreted in terms of association: categories with high coordinates and close spatial proximity indicate a direct association, whereas categories that are distant and exhibit opposite signs suggest an inverse relationship^17,25-26^.

#### Cluster analysis

Using the components derived from dimensionality reduction, a hierarchical clustering analysis was conducted employing Ward’s method. Both the MCA and the clustering analysis were performed using the FactoMineR package in R, version 4.2^27^.

### Data availability statement

The data are available in a data repository: Documentos y bases ELCA (https://datoscede.uniandes.edu.co/elca-ronda-3-2016/).

## RESULTS

ELCA provided longitudinal data on sociodemographic and employment variables for household heads in the years 2010, 2013, and 2016. As shown in Table 1, the population exhibited progressive aging across the analyzed period. In terms of marital status, there was a decrease in the proportion of individuals who were married or cohabiting, alongside an increase in those who were separated, divorced, or widowed. The majority of respondents consistently identified as heads of household and as white or mixed-race throughout the study period. From a sociodemographic perspective, the distribution across socioeconomic strata remained stable, with a slight improvement in access to basic public services. Access to telephone services showed a marginal increase; however, a substantial portion of the population continued to lack access to these essential resources. With respect to educational attainment, in 2010, only 17.2% of men and 18.1% of women reported having completed higher education, rising to 20.7% of men and 24.5% of women by 2016.

Self-reported CDs increased from 28.4 to 53.8% in men and from 35.8 to 63.3% in women over the study period (Table 1). The reported prevalence of a family history of CD remained relatively stable, with approximately 38.4% of men and 40.4% of women indicating such a history in their paternal line. These findings underscore the importance of considering family history in health risk assessments and highlight the need for preventive interventions.

In the labor market, variations are observed in the types of contracts and the economic sectors in which respondents are employed. Permanent contracts have declined, while verbal contracts and the “Does not have a contract” category have increased, particularly among women. This shift may indicate a trend toward informality in the labor market. Concurrently, educational attainment shows a decline in the completion of primary education, alongside an increase in higher education levels, suggesting a positive shift in human capital accumulation.

**Figure 1:**
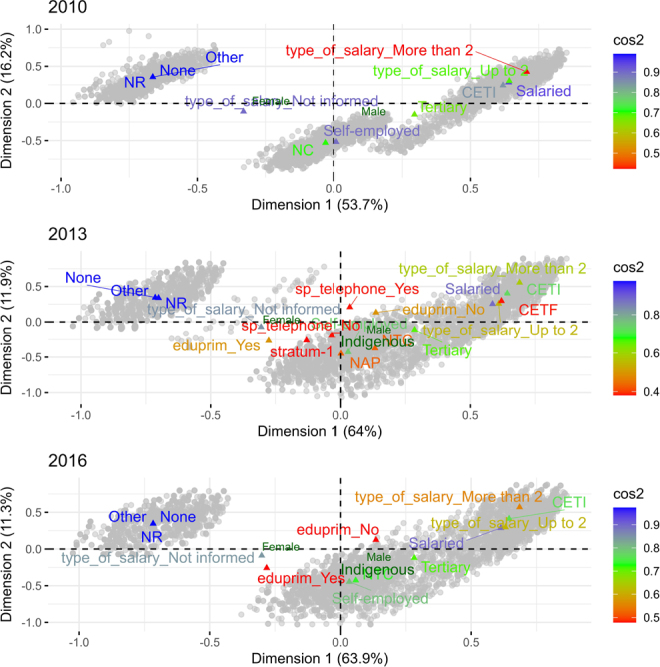
Correspondence analysis of each wave of the Colombian Longitudinal Survey.

Figure 1 presents the two-dimensional MCA maps for the periods under study. The dimensions are interpreted based on the contribution of each category to the total inertia, which represents the percentage of variability explained by each dimension. In 2010, the first two dimensions explained 69.9% of the total inertia, with 53.7% accounted for by the first dimension (x-axis) and 16.2% by the second (y-axis). In 2013, these two dimensions explained 75.9% of the inertia, with 64% attributed to the first dimension and 11.9% to the second. Similarly, in 2016, the first two dimensions explained 75.2% of the inertia, with 63.9% attributed to the first dimension and 11.3% to the second.

In this study, only those variables with squared cosine values greater than 0.4 were selected for representation in the graphic. This indicator quantifies the fidelity with which each variable is projected onto an axis or principal component. The cos^2^ values — represented using a color scale — enhance the visualization of variables within the analyzed dimensions. For example, in 2010, there is a close association between categories related to wage type and employment status (“type_of_salary_ LMMW” and “Salaried”), as reflected by their proximity in the upper right quadrant of the graphic. In contrast, the economic sector “None” and the unreported contract type (“NR”) suggest differentiated associations regarding the economic conditions of the respondents. Over time, some categories maintain a consistent position, such as the “Self-employed” work category, which is consistently located in the lower right quadrant throughout the three periods studied. On the other hand, other categories, such as the use of telephone services (“sp_telephone_yes”), show greater dispersion in the years 2013 and 2016, highlighting variations in their relationships with other variables. In these two-dimensional maps, gender has been included as a supplementary variable, projected onto the two-dimensional map; in the context of this type of analysis, such variables are projected later onto the factorial planes. In this case, a notable pattern is observed: the two levels of gender are positioned oppositely, with men (M) occupying the two right quadrants and women (W) the left quadrants.

**Figure 2:**
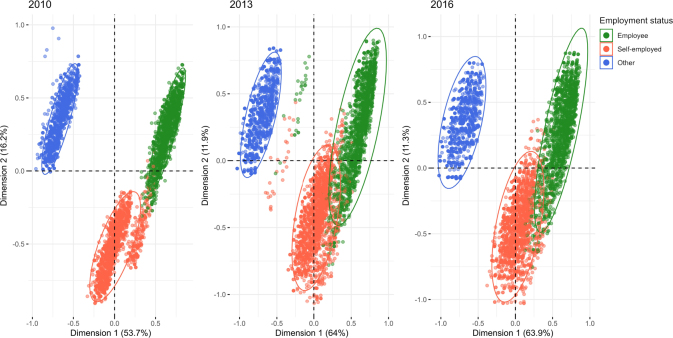
Correspondence analysis based on employment conditions in each wave of the ELCA.

Figure 2 presents the results of the hierarchical cluster analysis. Salary status was the key variable used to differentiate the sample into three groups: formal, semi-informal, and informal employment. The ellipses surrounding the clusters represent areas of concentration for each salary category. Across the three study periods, changes in the density and relative position of these clusters are observed, potentially indicating shifts in the labor structure or in the characteristics of jobs associated with each salary category. Subsequently, the variables corresponding to each group identified in Figure 2 were descriptively analyzed, as shown in Table 2.

**Table 2:** Statistics* of the groups separated by the cluster analysis derived from the MCA.

Year	2010				2013				2016			
Cluster	1	2	3	p.overall	1	2	3	p.overall	1	2	3	p.overall
	*N=1,725*	*N=1,927*	*N=1,981*		*N=1,608*	*N=2,606*	*N=1,419*		*N=1,581*	*N=2,253*	*N=1,799*	
**Chronic Illness or Pain**												
*Without CI*	1,081 62.7%	1,267 65.7%	1,438 72.6%	<0.001	709 44.1%	1,289 49.5%	826 58.2%	<0.001	518 32.8%	906 40.2%	862 47.9%	<0.001
*With CI*	644 37.3%	660 34.3%	543 27.4%		899 55.9%	1,317 50.5%	593 41.8%		1,063 67.2%	1,347 59.8%	937 52.1%	
**Type of Employment Relationship**												
*Salaried*	0 0.0%	0 0.0%	1,762 88.9%		27 1.7%	356 13.7%	1,398 98.5%		0 0.0%	29 1.3%	1,689 93.9%	<0.001
*Self-employed*	0 0.0%	1,927 100%	219 11.1%		27 1.7%	2,250 86.3%	21 1.5%		0 0.0%	2,224 98.7%	110 6.1%	
*Other*	1,725 100%	0 0.0%	0 0.0%		1,554 96.6%	0 0.0%	0 0.0%		1,581 100%	0 0.0%	0 0.0%	
**Salary Level**												
*Less than one* LMMW	0 0.0%	21 1.1%	601 30.3%	<0.001	0 0.0%	417 16.0%	348 24.5%	<0.001	0 0.0%	218 9.68%	458 25.5%	<0.001
*Up to 2* LMMW	0 0.0%	4 0.2%	993 50.1%		0 0.0%	159 6.10%	632 44.5%		0 0.0%	13 0.6%	846 47.0%	
*More than 2* LMMW	0 0.0%	0 0.0%	387 19.5%		0 0.0%	0 0.0%	0 0.0%		0 0.0%	0 0.0%	0 0.0%	
*Unknown salary*	1,725 100%	1,902 98.7%	0 0.0%		1,608 100%	1,981 76.0%	0 0.0%		1,581 100%	2,019 89.6%	45 2.5%	
**Type of Contract**												
*Written fixed-term contract*	0 0.0%	25 1.3%	330 16.7%	<0.001	0 0.0%	85 3.3%	362 25.5%	<0.001	0 0.0%	22 0.9%	470 26.1%	<0.001
*Written indefinite-term contract*	0 0.0%	0 0.0%	1,651 83.3%		0 0.0%	143 5.5%	776 54.7%		0 0.0%	20 0.9%	947 52.6%	
*Verbal*	0 0.0%	115 5.9%	0 0.0%		0 0.0%	152 5.8%	119 8.4%		0 0.0%	139 6.2%	181 10.1%	
*Not applicable*	0 0.0%	176 9.1%	0 0.0%		0 0.0%	1,106 42.4%	22 1.5%		0 0.0%	93 4.1%	0 0.0%	
*Not reported*	1,725 100%	0 0.0%	0 0.0%		1,608 100%	0 0.0%	0 0.00%		1,581 100%	0 0.0%	0 0.0%	
*No contract*	0 0.0%	1,611 83.6%	0 0.0%		0 0.0%	1,120 43.0%	140 9.8%		0 0.0%	1,979 87.8%	201 11.2%	
**Economic Sector**												
*None*	1,725 100%	0 0.0%	0 0.0%	<0.001	1,608 100%	0 0.0%	0 0.0%	<0.001	1,581 100%	0 0.0%	0 0.0%	<0.001
*Primary*	0 0.0%	84 4.4%	206 10.4%		0 0.0%	227 8.7%	64 4.5%		0 0.0%	135 6.0%	115 6.4%	
*Secondary*	0 0.0%	445 23.1%	415 20.9%		0 0.0%	517 19.8%	305 21.5%		0 0.0%	345 15.3%	327 18.2%	
*Tertiary*	0 0.0%	1,398 72.5%	1,360 68.7%		0 0.0%	1,862 71.5%	1,050 74.0%		0 0.0%	1,773 78.7%	1,357 75.4%	

*Weighted estimates of the sample. P-values obtained from the Pearson’s χ^2^ test for group comparisons. A p-value < 0.05 is considered statistically significant.

CI: Chronic Illness

LMMW:Legal Minimum Monthly Wage

The groups representing the different employment conditions Self-employed, Salaried, and Others were numerically designated as 1, 2, and 3, respectively. Changes in the composition of these groups over time are shown along with probability values (P) to determine whether the observed differences are statistically significant. At the top of the table, the presence of chronic disease is shown, with a general trend of increase in each group over time (p<0.001). This aspect requires further in-depth study to rule out age-related factors.

The type of employment contract also changes significantly over time within the groups (p=0.0001). There is a trend of a decreasing number of fixed-term written contracts and an increase in the “No contract” category, which could indicate job precarization. Finally, the economic sector in which individuals work shows significant variability (p=0.0001), with a notable decrease in the primary sector and an increase in the tertiary sector. This is consistent with a transition from an economy based on the production of raw materials to one focused on services.

## DISCUSION

This study assessed the prevalence of CD and their relationship with socioeconomic and occupational factors in Colombia using longitudinal survey data and MCA. The findings also suggest that working conditions may play a significant role in the development of CD. The observed increase in CD prevalence underscores the importance of socioeconomic determinants of health, as supported by previous studies^28,29,30^. Accordingly, this research aimed to inform public policy in Colombia, particularly regarding the extension of working life trajectories.

The increase in reported cases of CD may be attributed not only to biological or genetic factors, but also to evolving socioeconomic conditions. Changes in the distribution of employment contract types suggest a shift toward informal labor, which may adversely affect individuals’ health.

Differences in the prevalence of CD between men and women may be influenced by gender-specific roles and societal expectations, highlighting the importance of adopting gender-sensitive approaches in health research and policy^30^.

To advance the multidimensional analysis of categorical variables, MCA was employed, a technique well-suited for exploring latent relationships among qualitative variables by emphasizing the relative contribution of each category through cos^2^. Prior research, such as the analysis of the Spanish National Health Survey^24^, have demonstrated the utility of MCA in evaluating interactions between socioeconomic determinants and health profiles^31^. Consistent with these findings, MCA in the present study revealed associations among chronic comorbidities, reinforcing the importance of prioritizing categories with high explanatory inertia^31^. In this context, MCA enabled the graphical projection of 18 categorical variables in a two-dimensional space, illustrating the differential influence of occupational and socioeconomic factors on health trajectories through cos^2^, aligning with recent evidence on the multidimensional nature of social determinants^32^.

The integration of MCA with clustering techniques demonstrated its utility in capturing temporal dynamics, with the labor component emerging as a key determinant (cos^2^≥0.4), supporting previous hypotheses that link precarious employment to increased morbidity and mortality^33^. Nonetheless, certain methodological limitations must be acknowledged. MCA is sensitive to underrepresented categories (frequency <5%), which often exhibit marginal cos^2^ values (<0.10), leading to their underestimation in graphical representations. Moreover, the dominance of high-frequency categories in the total inertia may obscure the influence of less common variables, potentially underestimating important associations. This limitation can affect the comprehensiveness of the analysis and may restrict the generalizability of the findings to the broader population.

The findings indicate changes in the prevalence of CD, employment relationships, income levels, contract types, and economic sectors over the six-year period analyzed. The observed shifts in contract types and economic sector distribution suggest a trend toward increased labor informality, a phenomenon that, according to data from a systematic review, may negatively affect individual health outcomes^8^.

The association between labor informality and health determinants is consistent with findings from previous studies. For instance, research on diabetes prevalence in India highlights the influence of social determinants such as wealth and education^5^. Employment type has a direct impact on health, with individuals in informal employment being more vulnerable to CDs, a trend corroborated by the present study. Gaulke^34^ further emphasizes the impact of chronic diseases on the labor market, affecting not only individuals but also their families.

These findings are consistent with evidence indicating a correlation between the prevalence of CDs and changes in working conditions, including informality and job instability^21^. This association is particularly relevant in the Colombian context, where the transition toward informal employment and the variability in employment contracts may be contributing to the rising prevalence of CD.

The use of cluster analysis to segment the sample into groups may impose an artificial structure, potentially failing to capture the complexity and natural overlap between employment categories and income levels. Additionally, the accuracy of the employment and salary classifications in reflecting actual labor market conditions is limited, as is the presence of hidden heterogeneity within the “Other” and “Not Reported” categories. Furthermore, potential selection bias cannot be ruled out, as individuals who remain in the sample over time may not be representative of the original population or of the broader population, potentially leading to misinterpretation of observed trends. The data used for the MCA derive from a cluster sample, which relies on lottery-based selection and unit weights; thus, the findings are only applicable to the sample studied and cannot be generalized to the entire population.

The absence of detailed longitudinal information for each individual limits the capacity to establish causal relationships and fully understand individual work/health trajectories. While most MCA applications are cross-sectional, this study incorporates three time points, which enhances its temporal perspective. Similar approaches have been employed in studies by Bisquera et al.^35^ and Busija et al.^36^. However, to more robustly capture temporal effects, it is necessary to employ statistical techniques that account for autocorrelation inherent in longitudinal data, such as generalized estimating equations (GEE) or mixed-effects models. Accordingly, the findings should be interpreted with caution, as indicative rather than conclusive, regarding trends in employment and health conditions among the groups analyzed.

This study underscores the importance of incorporating working and socioeconomic conditions into public health analyses related to CDs. For health policies to be effective, they must be attuned to the specific characteristics of different employment contexts. Additionally, there is a need for more in-depth analyses employing techniques such as MCA and SCA), which facilitate a better understanding of the complex interactions between health outcomes and socioeconomic determinants.
