# Supplementary material for: Socioeconomic and labor characterization and prevalence of chronic disease in the Colombian population in the periods 2010, 2013, and 2016: A multiple correspondence analysis
Source: Rev Bras Epidemiol. 2025 Jul 4;28:e250036. doi: 10.1590/1980-549720250036 (PMC12227103; doi:10.1590/1980-549720250036)
Supplement: Translation

# Translation: Caracterización socioeconómica y laboral y prevalencia de enfermedad crónica en la población colombiana en los períodos 2010, 2013 y 2016: un análisis de correspondencias múltiples

**DOI:** 10.1590/1980-549720250036.2

**Authors:** Francisco Palencia-Sánchez, Gustavo Antonio Bruges Morales, Martha Riaño-Casallas

**Affiliations:** I Pontificia Universidad Javeriana, School of Medicine – Bogotá, Colombia.; II Universidad Autónoma de Bucaramanga, School of Health Sciences – Floridablanca, Santander, Colombia.; III Universidad Nacional de Colombia, School of Economic Sciences – Bogotá, Colombia.

**Keywords:** Determinantes sociales de la salud, Enfermedad crónica, Condiciones de trabajo, Salud pública, Análisis multivariante, Factores socioeconómicos

## Abstract

**Objetivos:**

Analizar la relación entre la prevalencia de enfermedades crónicas y los determinantes socioeconómicos, demográficos y laborales en la población colombiana en los años 2010, 2013 y 2016. Se buscó identificar patrones de asociación entre estas variables y evaluar cómo han evolucionado a lo largo del tiempo, con un énfasis particular en las implicaciones para la salud pública, especialmente en contextos de trabajo informales.

**Métodos:**

Se utilizan datos de la Encuesta Longitudinal Colombiana Urbana, que abarca estratos socioeconómicos de 1 a 4. La muestra incluye tanto a hombres como mujeres y a jefes de hogar y cónyuges, con edades comprendidas entre los 18 y 65 años. Las variables de interés se clasifican en tres categorías: salud, factores laborales y sociodemográficos. La metodología principal empleada es el Análisis de Correspondencia Múltiple (MCA).

**Resultados:**

Se identificaron tres grupos laborales diferenciados en la muestra: formal, semi-formal e informal. El grupo de trabajadores informales mostró una mayor prevalencia de enfermedades crónicas en comparación con los otros dos grupos. La categoría laboral fue el determinante social de mayor relevancia en la variabilidad de la salud.

**Conclusiones:**

El estudio revela que la informalidad laboral está asociada con una mayor susceptibilidad a enfermedades crónicas en Colombia. Estos resultados subrayan la necesidad de intervenciones políticas enfocadas en mejorar las condiciones laborales como una estrategia para mejorar la salud pública.

## INTRODUCCIÓN

La interacción entre el estado de salud de los trabajadores y su desempeño laboral es un elemento que afecta tanto la productividad como las dinámicas del sistema de salud y pensiones en el mundo. Esto ha motivado numerosos estudios sobre la población económicamente activa (PEA), con un enfoque particular en las enfermedades crónicas (EC), definidas por la Organización Mundial de la Salud^1^ como afecciones de larga duración resultantes de una combinación de factores genéticos, fisiológicos, ambientales y de comportamiento.

Estudios revelan que un porcentaje significativo de la población trabajadora padece una o más EC, impactando directamente en las decisiones relacionadas con las pensiones de discapacidad; se ha documentado como pueden disminuir la capacidad de trabajar entre un 17.8 al 36.4%^2^. Se ha observado que afecciones como las enfermedades mentales, cardiovasculares y musculoesqueléticas son causas frecuentes de retiro anticipado del mercado laboral. En Finlandia trabajadores entre los 35 y 54 años por enfermedades mentales presentan 4889 casos de pensiones de invalidez^3^. Este hecho, sumado al efecto del trabajador sano, dificulta una exploración más profunda sobre las EC en el contexto laboral. El efecto del trabajador sano se define como el fenómeno donde los trabajadores suelen exhibir menores tasas de mortalidad que la población general, ya que quienes padecen enfermedades severas o discapacidad son generalmente excluidos del empleo^4^. Por lo tanto, la aparente menor prevalencia de ciertas enfermedades entre trabajadores activos se debe principalmente a un sesgo de selección. Este efecto enmascara en parte la verdadera relación entre trabajo y EC, limitando la investigación al respecto.

Tradicionalmente, el impacto de las EC se ha examinado desde la perspectiva del sistema de salud, involucrando a actores que van desde clínicos y aseguradores hasta decisores de políticas públicas. Sin embargo, es fundamental ampliar este enfoque para comprender mejor las implicaciones de las EC en el ámbito laboral revisando los determinantes sociales de la salud en particular el trabajo^5^.

La dinámica laboral global muestra un aumento en la proporción de trabajadores entre 55 a 64 años, y varía según el sector económico teniendo implicaciones significativas en la salud laboral^6^. Así desde la política pública se han propuesto iniciativas para la inclusión de trabajadores de mayor edad quienes son los que más riesgos tienen de padecer una EC^7,8^.

En Colombia existen datos que evidencian que la principal afectación a la salud de los trabajadores se debe a la enfermedad general y no a la laboral. La enfermedad general puede entenderse como una consecuencia de una enfermedad o accidente de origen común que no está relacionada con la exposición ocupacional^9^. Según la Asociación Nacional de Industriales, sobre el ausentismo en empresas el 65.3% de los casos fueron originados por enfermedad general^10^. Estos datos, referentes solo a trabajadores formales, subrayan la importancia de considerar las diferentes categorías de empleo (asalariados, no asalariados, informales) y su relación con las EC.

Se ha observado en el mundo un aumento de la PEA en años recientes. Así, en Estados Unidos la proporción de trabajadores entre 55 y 64 años está creciendo más rápidamente que otros grupos etarios dentro de la fuerza laboral, una dinámica que puede variar según el sector económico^11^.

En Colombia, el agotamiento del “bono demográfico” y el incremento de personas mayores de 40 años en la PEA plantean desafíos relacionados con la transición demográfica y epidemiológica, lo cual podría influir en la incidencia y prevalencia de las EC, afectando así los costos del sistema de salud^12-14^. Con relación a las causas de muertes en Colombia principalmente se asocian a EC como enfermedad cardiovascular, enfermedad pulmonar obstructiva, accidente cerebrovascular en 2021 de acuerdo con un estudio de la principal casusa de muerte en el mundo^15^. De acuerdo con un informe del Banco de la República de Colombia, la salud de los trabajadores se ve afectada principalmente por enfermedades generales y no tanto por afecciones laborales^16^.

La EC es multifactorial, por tanto, el análisis de correspondencias múltiples (MCA) permite comprender esta complejidad detectando patrones homogéneos^17^. El propósito de esta investigación es analizar la prevalencia de EC y su relación con variables socioeconómicas y laborales en una muestra de personas seguidas durante 2010, 2013 y 2016 en Colombia mediante un MCA.

## MÉTODOS

### Fuente de los datos

Se utilizó la Encuesta Longitudinal Colombiana (ELCA) en su componente urbano, que realizó un seguimiento longitudinal a 6000 hogares desde 2010, y dos evaluaciones adicionales en 2013 y 2016. Dicha encuesta diseñada por el Centro de Estudios sobre Desarrollo Económico (CEDE)^18^ de la Facultad de Economía de la Universidad de los Andes de Colombia permitió recolectar información sobre cambios temporales en dinámicas que afectan el bienestar de la población. La muestra, representativa de los estratos socioeconómicos 1 a 4 a nivel nacional y de cinco regiones geográficas de Colombia, es de carácter probabilístico, estratificado, polietápico y por conglomerados^10^.

La relevancia de este estudio reside en su naturaleza longitudinal, lo cual responde a la necesidad en Colombia de consolidar investigaciones prospectivas sobre poblaciones clave en la toma de decisiones en política pública. En este caso, la estrategia consistió en separar al mismo grupo de individuos en los tres momentos de aplicación de la encuesta.

### Metodología

El estudio se centró en población económicamente activa, definida como aquella constituida por individuos en edad laboral que están ocupados y/o en búsqueda de empleo. Para el análisis, se seleccionaron jefes de hogar de 18 a 65 años en la línea base, excluyendo a menores de 18 años para evitar confusión con el trabajo infantil^19-21^.

Las variables examinadas abarcan aspectos de salud, laborales y sociodemográficos como se muestra en la Tabla 1. Para garantizar la fiabilidad y validez del presente estudio se consideraron como variables de confusión: el *estrato socioeconómico* ya que afecta tanto a las oportunidades de empleo como a la accesibilidad a servicios de salud. Similarmente, la *edad* debido a su influencia en la elección del sector de empleo y en la prevalencia de EC. Por último, la *región* ya que puede incidir tanto en las oportunidades educativas, como en las condiciones económicas regionales.

**Tabla 1:** Resumen descriptivo (*) de las variables estudiadas de la encuesta ELCA para los periodos 2010, 2013 y 2016.

Año	2010		2013		2016	
Variable	hombres	mujeres	hombres	mujeres	hombres	mujeres
	*n=2286*	*n=3347*	*n=2286*	*n=3347*	*n=2286*	*n=3347*
**Auto reporte de enfermedad o dolor crónico**						
*No*	1636 (71.6%)	2150 (64.2%)	1264 (55.3%)	1560 (46.6%)	1057 (46.2%)	1229 (36.7%)
*Sí*	650 (28.4%)	1197 (35.8%)	1022 (44.7%)	1787 (53.4%)	1229 (53.8%)	2118 (63.3%)
**Edad (años)**						
*18-34*	560 (24.5%)	1003 (30.0%)	393 (17.2%)	730 (21.8%)	240 (10.5%)	478 (14.3%)
*35-50*	1033 (45.2%)	1532 (45.8%)	996 (43.6%)	1533 (45.8%)	958 (41.9%)	1474 (44.0%)
*51-71*	693 (30.3%)	812 (24.3%)	897 (39.2%)	1084 (32.4%)	1088 (47.6%)	1395 (41.7%)
**Estado civil**						
*En unión libre*	1016 (44.4%)	1134 (33.9%)	1072 (46.9%)	1167 (34.9%)	1098 (48.0%)	1176 (35.1%)
*Separado(a) o divorciado(a)*	1100 (48.1%)	1277 (38.2%)	1000 (43.7%)	1137 (34.0%)	941 (41.2%)	1054 (31.5%)
*Soltero(a)*	84 (3.7%)	511 (15.3%)	125 (5.5%)	603 (18.0%)	153 (6.7%)	624 (18.6%)
*Viudo(a)*	75 (3.3%)	269 (8.0%)	71 (3.1%)	266 (7.9%)	69 (3.0%)	286 (8.5%)
*En unión libre*	11 (0.5%)	152 (4.5%)	18 (0.8%)	174 (5.2%)	25 (1.1%)	207 (6.2%)
*No informa*	0 (0.0%)	4 (0.1%)	0 (0.0%)	0 (0.0%)	0 (0.0%)	0 (0.0%)
**Posición en el hogar**						
*Jefe de hogar*	2080 (91.0%)	1259 (37.6%)	2080 (91.0%)	1259 (37.6%)	2080 (91.0%)	1259 (37.6%)
*Cónyuge*	206 (9.0%)	2088 (62.4%)	206 (9.0%)	2088 (62.4%)	206 (9.0%)	2088 (62.4%)
**Región del país**						
*Atlántica*	604 (26.4%)	816 (24.4%)	604 (26.4%)	816 (24.4%)	604 (26.4%)	816 (24.4%)
*Bogotá*	331 (14.5%)	509 (15.2%)	331 (14.5%)	509 (15.2%)	331 (14.5%)	509 (15.2%)
*Central*	405 (17.7%)	648 (19.4%)	405 (17.7%)	648 (19.4%)	405 (17.7%)	648 (19.4%)
*Oriental*	473 (20.7%)	691 (20.6%)	473 (20.7%)	691 (20.6%)	473 (20.7%)	691 (20.6%)
*Pacífica*	473 (20.7%)	683 (20.4%)	473 (20.7%)	683 (20.4%)	473 (20.7%)	683 (20.4%)
**Grupo étnico**						
*Afrocolombiano*	225 (9.8%)	275 (8.2%)	225 (9.8%)	275 (8.2%)	225 (9.8%)	275 (8.2%)
*Blanco-Mestizo*	1930 (84.4%)	2879 (86.0%)	1930 (84.4%)	2879 (86.0%)	1930 (84.4%)	2879 (86.0%)
*Indígena*	131 (5.7%)	193 (5.8%)	131 (5.7%)	193 (5.8%)	131 (5.7%)	193 (5.8%)
**Estrato socio económico de la vivienda que habita**						
*estrato-1*	745 (32.6%)	1019 (30.4%)	748 (32.7%)	1038 (31.0%)	723 (31.6%)	1010 (30.2%)
*estrato-2*	912 (39.9%)	1361 (40.7%)	907 (39.7%)	1344 (40.2%)	931 (40.7%)	1353 (40.4%)
*estrato-3*	534 (23.4%)	834 (24.9%)	547 (23.9%)	842 (25.2%)	526 (23.0%)	836 (25.0%)
*estrato-4*	95 (4.2%)	133 (4.0%)	84 (3.7%)	123 (3.7%)	106 (4.6%)	148 (4.4%)
**Nivel educativo**						
*Primaria*						
*No*	1397 (61.1%)	2095 (62.6%)	1503 (65.7%)	2271 (67.9%)	1511 (66.1%)	2293 (68.5%)
*Sí*	889 (38.9%)	1252 (37.4%)	783 (34.3%)	1076 (32.1%)	775 (33.9%)	1054 (31.5%)
*Secundaria*						
*No*	1330 (58.2%)	1918 (57.3%)	1325 (58.0%)	1933 (57.8%)	1333 (58.3%)	1976 (59.0%)
*Sí*	956 (41.8%)	1429 (42.7%)	961 (42.0%)	1414 (42.2%)	953 (41.7%)	1371 (41.0%)
*Superior*						
*No*	1892 (82.8%)	2741 (81.9%)	1817 (79.5%)	2573 (76.9%)	1813 (79.3%)	2528 (75.5%)
*Sí*	394 (17.2%)	606 (18.1%)	469 (20.5%)	774 (23.1%)	473 (20.7%)	819 (24.5%)
**Tipo de relación laboral**						
*Cuenta Propia*	1060 (46.4%)	1086 (32.4%)	1063 (46.5%)	1235 (36.9%)	1085 (47.5%)	1249 (37.3%)
*Asalariado*	986 (43.1%)	776 (23.2%)	979 (42.8%)	802 (24.0%)	898 (39.3%)	820 (24.5%)
*Otro*	240 (10.5%)	1485 (44.4%)	244 (10.7%)	1310 (39.1%)	303 (13.3%)	1278 (38.2%)
**Valor del salario**						
*No informa*	1215 (53.1%)	2412 (72.1%)	1534 (67.1%)	2543 (76.0%)	1587 (69.4%)	2511 (75.0%)
*Bajo SMMLVA*	227 (9.9%)	395 (11.8%)	282 (12.3%)	483 (14.4%)	224 (9.8%)	452 (13.5%)
*Hasta 2 SMMLVA*	603 (26.4%)	394 (11.8%)	470 (20.6%)	321 (9.6%)	475 (20.8%)	384 (11.5%)
*Mas 2 SMMLVA*	241 (10.5%)	146 (4.4%)	0 (0.0%)	0 (0.0%)	0 (0.0%)	0 (0.0%)
**Tipo de contrato**						
*Escrito a término fijo*	106 (4.6%)	249 (7.4%)	254 (11.1%)	193 (5.8%)	236 (10.3%)	256 (7.6%)
*Escrito a término indefinido*	965 (42.2%)	686 (20.5%)	531 (23.2%)	388 (11.6%)	537 (23.5%)	430 (12.8%)
*Verbal*	91 (4.0%)	24 (0.7%)	129 (5.6%)	142 (4.2%)	150 (6.6%)	170 (5.1%)
*No aplica*	61 (2.7%)	115 (3.4%)	497 (21.7%)	631 (18.9%)	37 (1.6%)	56 (1.7%)
*No tiene*	823 (36.0%)	788 (23.5%)	606 (26.5%)	654 (19.5%)	1023 (44.8%)	1157 (34.6%)
*NR*	240 (10.5%)	1485 (44.4%)	269 (11.8%)	1339 (40.0%)	303 (13.3%)	1278 (38.2%)
**Sector Económico donde trabaja**						
*Ninguno*	240 (10.5%)	1485 (44.4%)	269 (11.8%)	1339 (40.0%)	303 (13.3%)	1278 (38.2%)
*Primario*	232 (10.1%)	58 (1.7%)	237 (10.4%)	54 (1.6%)	204 (8.9%)	46 (1.4%)
*Secundario*	540 (23.6%)	320 (9.6%)	510 (22.3%)	312 (9.3%)	459 (20.1%)	213 (6.4%)
*Terciario*	1274 (55.7%)	1484 (44.3%)	1270 (55.6%)	1642 (49.1%)	1320 (57.7%)	1810 (54.1%)
**Servicio Público Gas**						
*No tiene*	699 (30.6%)	1032 (30.8%)	509 (22.3%)	750 (22.4%)	385 (16.8%)	567 (16.9%)
*Sí tiene*	1587 (69.4%)	2315 (69.2%)	1777 (77.7%)	2597 (77.6%)	1901 (83.2%)	2780 (83.1%)
**Servicio Público Teléfono**						
*No tiene*	1134 (49.6%)	1640 (49.0%)	1184 (51.8%)	1727 (51.6%)	1251 (54.7%)	1807 (54.0%)
*Sí tiene*	1152 (50.4%)	1707 (51.0%)	1102 (48.2%)	1620 (48.4%)	1035 (45.3%)	1540 (46.0%)
**Enfermedad por parte de padre**						
No	1409 (61.6%)	1994 (59.6%)	1409 (61.6%)	1994 (59.6%)	1409 (61.6%)	1994 (59.6%)
Sí	877 (38.4%)	1353 (40.4%)	877 (38.4%)	1353 (40.4%)	877 (38.4%)	1353 (40.4%)
**Enfermedad por parte de madre**						
*No*	1166 (51.0%)	1629 (48.7%)	1166 (51.0%)	1629 (48.7%)	1166 (51.0%)	1629 (48.7%)
*Sí*	1120 (49.0%)	1718 (51.3%)	1120 (49.0%)	1718 (51.3%)	1120 (49.0%)	1718 (51.3%)

*Estimaciones ponderadas de la muestra.

Cada observación obtenida de la encuesta incluye un factor de expansión para garantizar su representatividad estadística. Estos factores se utilizaron directamente como pesos en el cálculo de proporciones y posteriores análisis mediante el paquete survey de R. Para detalles metodológicos, consulte la guía del usuario (https://datoscede.uniandes.edu.co/wp-content/uploads/Guia-Usuario.pdf)^22^.

### Estadística descriptiva

Se elaboró una tabla para presentar las frecuencias absolutas y porcentajes de las variables categóricas. Esto incluye la evaluación de la prevalencia de EC, la distribución de tipos de contrato y sectores económicos, así como datos sociodemográfica como el nivel educativo, el estrato socioeconómico y etnia, diferenciados por cada año del estudio (2010, 2013 y 2016). Las tablas descriptivas fueron elaboradas con el paquete compareGroups de R^23^.

Para los cálculos, se emplearon las funciones del paquete survey de R, que gestiona automáticamente los factores de expansión como pesos estadísticos “survey: analysis of complex survey samples.” R package version 4.4)^24^.

### Análisis de correspondencia múltiple

El MCA es una técnica utilizada para visualizar patrones de datos categóricos de gran tamaño y alta dimensionalidad^17^. En el MCA, se puede hacer una interpretación midiendo la contribución de las variables a cada uno de los ejes, donde se consideran medidas varias medidas como la inercia, los valores propios y el coseno cuadrado (cos^2^). En este estudio se destaca el empleo del cos^2^ para evaluar la calidad de la representación gráfica, facilitando así la identificación de aquellas variables que se proyectan de manera óptima en el espacio factorial. En el MCA, la distancia entre las categorías de distintas variables se interpreta en términos de asociación. Dos categorías con coordenadas elevadas y se ubican próximas en el espacio indican una asociación directa; por el contrario, si se encuentran separadas —exhibiendo signos opuestos— se infiere la existencia de una relación inversa^17,25-26^.

#### Análisis de clúster

Con las componentes de reducción de la dimensionalidad se procedió a desarrollar un análisis de agrupamiento jerárquico utilizando el método de Ward. Tanto el MCA como el análisis de Clúster fueron realizados con el paquete FactoMineR en R, versión4.2^27^.

### Declaración de disponibilidad de datos

Los datos están disponibles en el repositorio Documentos y bases ELCA https://datoscede.uniandes.edu.co/elca-ronda-3-2016/.

## RESULTADOS

La ELCA proporcionó datos longitudinales sobre variables sociodemográficas y laborales de jefes de hogar, para 2010, 2013 y 2016. La Tabla 1 describe que por grupos de edad la población muestra un envejecimiento progresivo en el periodo analizado. Con relación al estado civil, se ha registrado una disminución en la proporción de individuos casados o en unión libre y un incremento en aquellos separados, divorciados o viudos. La mayoría de los encuestados se identifican como jefes de hogar y blanco-mestizo, lo cual es coherente a lo largo de los años estudiados. Desde la perspectiva sociodemográfica, se mantiene una distribución estable en los estratos socioeconómicos, con una ligera mejoría en el acceso a servicios públicos básicos. La disponibilidad de servicios de telefonía muestra un incremento marginal, aunque una proporción significativa de la población sigue sin acceso a estos servicios esenciales. En cuanto al nivel educativo en 2010 solo el 17.2% de los hombres y el 18.1% de las mujeres manifestaron tener educación superior y en 2016 era el 20.7% de los hombres y 24.5% de las mujeres.

El autoreporte de EC aumenta del 28.4 al 53.8% en hombres y del 35.8 al 63.3% en mujeres durante el período estudiado (Tabla 1). La prevalencia de antecedentes de EC reportada permanece relativamente estable, con aproximadamente el 38.4% de los hombres y el 40.4% de las mujeres, indicando tales antecedentes en su línea paterna. Estos hallazgos resaltan la importancia de considerar la historia familiar en la evaluación de riesgos para la salud y la necesidad de intervenciones preventivas.

En el ámbito laboral, se observa una variación en los tipos de contrato y los sectores económicos en los que los encuestados están empleados. Los contratos a término indefinido han disminuido, mientras que los contratos verbales y la categoría de “No tiene” contrato han experimentado un aumento, especialmente entre las mujeres. Esta transición podría reflejar una evolución hacia la informalidad en el mercado laboral. Paralelamente, el nivel educativo muestra una disminución en la finalización de la educación primaria y un aumento en los niveles de educación superior, sugiriendo un cambio positivo en la acumulación de capital humano.

La Figura 1 muestra los mapas bidimensionales del MCA, correspondientes a los periodos estudiados. Las dimensiones se interpretan a partir de la contribución de cada categoría a la inercia total, la cual representa el porcentaje de variabilidad explicado por cada dimensión. En el año 2010, las dos primeras dimensiones explicaron el 69,9% de la inercia total, distribuyéndose un 53,7% en la primera dimensión (eje x) y un 16,2% en la segunda (eje y). En 2013, estas dos dimensiones abarcaron el 75,9% de la inercia, con una asignación del 64% a la primera dimensión y del 11,9% a la segunda. De manera similar, en 2016 las dos primeras dimensiones representaron el 75,2% de la inercia, destinándose un 63,9% a la primera dimensión y un 11,3% a la segunda.

**Figura 1:** Análisis de correspondencia de cada una de la ola de la Encuesta Longitudinal Colombiana.

En este estudio, se escogió representar en el gráfico exclusivamente aquellas variables que presentaron valores de coseno cuadrado superiores a 0,4. Este indicador permite cuantificar la fidelidad con la que cada variable se proyecta en un eje o componente principal. Los valores de cos^2^—visualizados mediante una escala cromática—refuerzan la representación de las variables en las dimensiones analizadas. Por ejemplo, en 2010, se observa una estrecha asociación entre las categorías vinculadas con el tipo de salario y la condición laboral (“tipo_de_salario_ SMMLVA” y “Asalariado”), como lo refleja su proximidad en el cuadrante superior derecho del gráfico; en contraste, el sector económico “Ninguno” y el tipo de contrato no reportado (“NR”) sugieren asociaciones diferenciadas con relación a las condiciones económicas de los entrevistados. A lo largo del tiempo, algunas categorías mantienen constante su posicionamiento, como es el caso de la categoría de trabajo “Cuenta propia”, consistentemente ubicada en el cuadrante inferior derecho durante los tres periodos estudiados. Por otro lado, otras categorías, como el uso de servicios telefónicos (“sp_telefono_Sí tiene”), muestran una mayor dispersión en los años 2013 y 2016, lo que pone de manifiesto las variaciones en sus relaciones con las demás variables. En estos mapas bidimensionales se ha incluido el sexo de los individuos como variable suplementaria, proyectada en el mapa bidimensional; en el contexto de este tipo de análisis estas variables se proyectan de manera posterior sobre los planos factoriales; destaca en este caso una ubicación de los dos niveles del sexo de manera opuesta, ocupando los hombres(H) los dos cuadrantes de la derecha y las mujeres (M) los cuadrantes izquierdos.

La Figura 2 muestra el análisis de clúster jerárquico. La variable que permitió una separación en tres grupos fue la de condición salarial, identificaron tres grupos laborales diferenciados en la muestra: formal, semi-informal e informal. Las elipses alrededor de los grupos de puntos sugieren áreas de concentración de los puntos para cada categoría salarial y a lo largo de los tres períodos estudiados se observan cambios en la densidad y la posición relativa de los grupos, lo que puede indicar cambios en la estructura laboral o en las características de los empleos asociados con cada categoría salarial. De esta manera se procedió a evaluar de manera descriptiva las variables para cada uno de los grupos derivados de la Figura 2 como se muestra en la Tabla 2.

**Figura 2:** Análisis de correspondencia teniendo en cuenta las condiciones de empleo en cada una de las olas de la ELCA.

**Tabla 2:** Estadísticas* de los grupos separados por el análisis de clúster derivado del MCA.

Año	2010				2013				2016			
Clúster	1	2	3	p.overall	1	2	3	p.overall	1	2	3	p.overall
	*N=1725*	*N=1927*	*N=1981*		*N=1608*	*N=2606*	*N=1419*		*N=1581*	*N=2253*	*N=1799*	
Dolor o enfermedad crónica												
*No EC*	1081 62.7%	1267 65.7%	1438 72.6%	<0.001	709 44.1%	1289 49.5%	826 58.2%	<0.001	518 32.8%	906 40.2%	862 47.9%	<0.001
*Si EC*	644 37.3%	660 34.3%	543 27.4%		899 55.9%	1317 50.5%	593 41.8%		1063 67.2%	1347 59.8%	937 52.1%	
**Tipo de relación laboral**												
*Asalariado*	0 0.0%	0 0.0%	1762 88.9%		27 1.7%	356 13.7%	1398 98.5%		0 0.0%	29 1.3%	1689 93.9%	<0.001
*Cuenta Propia*	0 0.0%	1927 100%	219 11.1%		27 1.7%	2250 86.3%	21 1.5%		0 0.0%	2224 98.7%	110 6.1%	
*Otro*	1725 100%	0 0.0%	0 0.0%		1554 96.6%	0 0.0%	0 0.0%		1581 100%	0 0.0%	0 0.0%	
**Nivel de salario**												
*Menor un SMMLVA*	0 0.0%	21 1.1%	601 30.3%	<0.001	0 0.0%	417 16.0%	348 24.5%	<0.001	0 0.0%	218 9.68%	458 25.5%	<0.001
*Hasta 2 SMMLVA*	0 0.0%	4 0.2%	993 50.1%		0 0.0%	159 6.10%	632 44.5%		0 0.0%	13 0.6%	846 47.0%	
*Mas 2 SMMLVA*	0 0.0%	0 0.0%	387 19.5%		0 0.0%	0 0.0%	0 0.0%		0 0.0%	0 0.0%	0 0.0%	
*Salario desconocido*	1725 100%	1902 98.7%	0 0.0%		1608 100%	1981 76.0%	0 0.0%		1581 100%	2019 89.6%	45 2.5%	
**Tipo de contrato**												
*Escrito término fijo*	0 0.0%	25 1.3%	330 16.7%	<0.001	0 0.0%	85 3.3%	362 25.5%	<0.001	0 0.0%	22 0.9%	470 26.1%	<0.001
*Escrito término indefinido*	0 0.0%	0 0.0%	1651 83.3%		0 0.0%	143 5.5%	776 54.7%		0 0.0%	20 0.9%	947 52.6%	
*Verbal*	0 0.0%	115 5.9%	0 0.0%		0 0.0%	152 5.8%	119 8.4%		0 0.0%	139 6.2%	181 10.1%	
*No aplica*	0 0.0%	176 9.1%	0 0.0%		0 0.0%	1106 42.4%	22 1.5%		0 0.0%	93 4.1%	0 0.0%	
*No reporta*	1725 100%	0 0.0%	0 0.0%		1608 100%	0 0.0%	0 0.00%		1581 100%	0 0.0%	0 0.0%	
*No tiene contrato*	0 0.0%	1611 83.6%	0 0.0%		0 0.0%	1120 43.0%	140 9.8%		0 0.0%	1979 87.8%	201 11.2%	
**Sector Económico**												
*Ninguno*	1725 100%	0 0.0%	0 0.0%	<0.001	1608 100%	0 0.0%	0 0.0%	<0.001	1581 100%	0 0.0%	0 0.0%	<0.001
*Primario*	0 0.0%	84 4.4%	206 10.4%		0 0.0%	227 8.7%	64 4.5%		0 0.0%	135 6.0%	115 6.4%	
*Secundario*	0 0.0%	445 23.1%	415 20.9%		0 0.0%	517 19.8%	305 21.5%		0 0.0%	345 15.3%	327 18.2%	
*Terciario*	0 0.0%	1398 72.5%	1360 68.7%		0 0.0%	1862 71.5%	1050 74.0%		0 0.0%	1773 78.7%	1357 75.4%	

*Estimaciones ponderadas de la muestra. Valores de P obtenidos del análisis χ^2^ de Pearson de comparación entre grupos. Un valor de p<0.05 es considerado estadísticamente significativo.

Los grupos de las diferentes condiciones Cuenta_Propia, Asalariados y Otros fueron designados numéricamente como 1, 2 y 3 respectivamente y los cambios de composición de estos grupos a lo largo del tiempo se muestran junto con los valores de probabilidad(P) para determinar si las diferencias observadas son estadísticamente significativas. En la parte superior de la tabla, se observa la presencia de enfermedad crónica, con una tendencia general a aumentar en cada grupo a lo largo del tiempo (p<0.001). Aspecto que necesita ser estudiado a profundidad para descartar factores asociados a la edad.

El tipo de contrato laboral también cambia significativamente con el tiempo dentro de los grupos (p=0.0001). Hay una tendencia de un número decreciente de contratos escritos a término fijo y un aumento en la categoría “No tiene contrato”, lo cual podría indicar una precarización del empleo. Finalmente, el sector económico donde trabajan los individuos muestra una variabilidad significativa (p=0.0001), con una disminución notable en el sector primario y un aumento en el sector terciario, lo cual es consistente con una transición de una economía basada en producción asociada materias primas a una orientada hacia servicios.

## DISCUSIÓN

Este estudio evaluó la prevalencia de EC y su relación con factores socioeconómicos y laborales en Colombia basados en una encuesta longitudinal utilizando el MCA. Además, muestra cómo las condiciones de trabajo podrían contribuir como un factor importante en la EC. El incremento en la prevalencia de EC resalta la relevancia de factores socioeconómicos en la salud, tal como se evidencia en trabajos previos^28-30^. Por tanto, esta investigación busca contribuir a la política pública en Colombia para la prolongación de la trayectoria de vida laboral.

Aumentos de la frecuencia en el reporte de EC podrían estar vinculados no solo a aspectos biológicos o genéticos, sino también a condiciones socioeconómicas cambiantes. Las variaciones en los tipos de contrato sugieren una transición hacia la informalidad laboral, lo cual a su vez podría impactar negativamente la salud de los individuos.

Las diferencias en la prevalencia de EC entre hombres y mujeres podrían estar influenciadas por roles y expectativas de género específicos, lo que subraya la importancia de adoptar enfoques en salud desde la perspectiva de género^30^.

Para profundizar en el análisis multidimensional de las variables categóricas, se implementó un MCA, técnica idónea para explorar relaciones latentes entre variables cualitativas, destacando la contribución relativa de cada categoría mediante el cos^2^. Estudios previos, como el análisis de la Encuesta Nacional de Salud de España^24^, han validado su utilidad en la evaluación de interacciones entre determinantes socioeconómicos y perfiles de salud^31^. En línea con esto, el MCA ha revelado asociación entre comorbilidades crónicas, subrayando la necesidad de priorizar categorías con alta inercia explicativa^31^. En nuestro estudio, la aplicación del MCA permitió proyectar gráficamente 18 variables categóricas en un espacio bidimensional, resaltando mediante el cos^2^ la influencia diferencial de factores laborales y socioeconómicos en las trayectorias de salud, lo que coincide con hallazgos recientes sobre la multidimensionalidad de los determinantes sociales^32^.

La integración del MCA con técnicas de clúster evidenció su evolución temporal, donde el componente laboral emergió como elemento determinante (cos2≥0.4) respaldando hipótesis previas sobre la precarización como predictor de morbimortalidad^33^. Sin embargo, se reconocen limitaciones metodológicas, el MCA presenta sensibilidad a categorías infrarrepresentadas (frecuencia <5%), las cuales, al exhibir cos^2^ marginales (<0.10), pueden subestimarse en la representación gráfica. Adicionalmente, la dominancia de las categorías de alta frecuencia en la inercia total puede relegar la influencia de aquellas menos frecuentes, subestimando relaciones potencialmente significativas y comprometiendo una visión integral de las interacciones entre las variables estudiadas, así como hacer la generalización a toda la población.

Los hallazgos sugieren cambios en la prevalencia de EC, la naturaleza de la relación laboral, el nivel de ingresos, el tipo de contrato y el sector económico a lo largo de un periodo de seis años. Las variaciones observadas en los tipos de contrato y en los sectores económicos apuntan una transición hacia la informalidad laboral, un fenómeno que podría tener un impacto negativo en la salud de los individuos tal como lo mencionan los datos de una revisión sistemática^8^.

La informalidad laboral y su asociación con determinantes de salud está en consonancia con estudios previos, por ejemplo, la prevalencia de diabetes en India donde se observaron determinantes sociales como el índice de riqueza y la educación^5^. El tipo de empleo tiene un impacto directo en la salud. Las personas en condiciones laborales informales, por ejemplo, son más susceptibles a EC, una tendencia que también se reflejó en esta investigación. Gaulke^34^ aborda cómo las EC impactan en el mercado laboral, tanto a nivel individual como familiar.

Esto concuerda con los hallazgos donde se observa una correlación entre la prevalencia de EC y cambios en las condiciones laborales, como la informalidad y la inestabilidad del empleo^21^. Esto es pertinente en el contexto colombiano, donde la transición hacia la informalidad laboral y la variabilidad en los contratos de trabajo podrían estar contribuyendo al aumento en la prevalencia de EC.

El uso del análisis clúster para segmentar la muestra en grupos también puede imponer una estructura artificial, no capturando la complejidad y solapamiento natural entre categorías laborales e ingresos. La precisión de las categorías de empleo y salario para reflejar la realidad es otra limitación, así como la heterogeneidad oculta en las categorías “Otros” y “No reporta”. Además, no se puede descartar un posible sesgo de selección, dado que los individuos que permanecen en cada grupo con el tiempo podrían no ser representativos de la población general o de los grupos al inicio del estudio, conduciendo a interpretaciones erróneas de las tendencias y cambios observados. Los datos utilizados para el MCA proceden de una muestra por conglomerados, que utiliza sorteos y ponderaciones unitarias. Por lo tanto, los resultados solo son aplicables a la muestra y no pueden generalizarse a la población estudiada.

La ausencia de información longitudinal detallada para cada individuo limita establecer causalidad y comprender trayectorias laborales/de salud individuales. La mayoría de los estudios de MCA se aplican en un momento determinado en el tiempo, aquí se logró obtener tres mediciones en el tiempo. Otros estudios donde se aplica esta misma aproximación son Bisquera et al.^35^ y Busija et al.^36^. Para evidenciar con mayor profundidad el efecto temporal, se requiere la aplicación de técnicas que controlen aspectos de autocorrelación de variables que pueden surgir en este tipo de estudios longitudinales, como los modelos de ecuaciones de estimación generalizadas (GEE) o los modelos de efectos mixtos. Por tanto, los resultados deben interpretarse con cautela y como indicativos, pero no definitivos, de las tendencias en condiciones laborales y de salud en los grupos estudiados.

Sugerimos la importancia de considerar las condiciones laborales y socioeconómicas en el análisis de la salud pública en lo que respecta a EC. Las políticas de salud deben ser sensibles a los contextos laborales específicos para ser efectivas. Además, resalta la necesidad de un análisis más profundo que incorpore técnicas como el MCA y SCA para para comprender las interacciones entre salud y factores socioeconómicos.
